# Prostate cancer clinical characteristics and outcomes in Central Sudan

**DOI:** 10.3332/ecancer.2021.1265

**Published:** 2021-07-08

**Authors:** Sami Mahjoub Taha, Hsin-Yi Weng, Mohammed El Imam Mohammed, Yassin M Osman, N’sanh N’dri, Sulma I Mohammed, Dafalla Omer Abuidris

**Affiliations:** 1 Urology Department, Gezira Hospital for Renal Disease and Surgery, PO Box 20, Sudan; 2 Department of Comparative Pathobiology and Purdue University Center for Cancer Research, Purdue University, West Lafayette, IN 47907, USA; 3 Oncology Department, National Cancer Institute, University of Gezira, Sudan

In the abstract, in the methodology section, line number 1, the following sentence: ‘This study was conducted prospectively at the Gezira Hospital for Renal Disease and Surgery and at the National Cancer Institute at the University of Gezira, Sudan, for an 11-year period’ should be replaced with ‘This study was conducted retrospectively at the Gezira Hospital for Renal Disease and Surgery and at the National Cancer Institute at the University of Gezira, Sudan, for an 11-year period.’

